# Editorial “Thermodynamic Optimization of Industrial Energy Systems”

**DOI:** 10.3390/e26121047

**Published:** 2024-12-03

**Authors:** Daniel Florez-Orrego, Meire Ellen Ribeiro Domingos, Rafael Nogueira Nakashima

**Affiliations:** 1 Industrial Process and Energy Systems Engineering, École Polytechnique Fédérale de Lausanne, 1950 Sion, Valais, Switzerland; meire.ribeirodomingos@epfl.ch; 2 Faculty of Mines, National University of Colombia, Av. 80 #65–223, Medellin 1779, Antioquia, Colombia; 3 Department of Energy Conversion and Storage, Technical University of Denmark DTU, 2800 Kongens Lyngby, Denmark; rafnn@dtu.dk

Thermodynamic optimization of industrial energy systems is crucial for finding solutions to reduce energy consumption and mitigate losses, leading to environmental and economic benefits. It involves applying thermodynamic principles to enhance the performance of the industrial, chemical and power generation systems, from individual components to entire plants. Among the widely used techniques, heat integration (i.e., the pinch method) and exergy analysis stand out for their ability to pinpoint sources of inefficiency [1]. However, those systems are part of a larger ecosystem, involving complex exchanges and interactions with other energy systems, and with the society and the environment. For this reason, process integration and thermodynamic optimization methods have been extended from a classical perspective of waste heat recovery to include a holistic design and optimization approach. In fact, a systemic dimension of process integration can cover aspects like (i) heat pumps and electrification; (ii) carbon capture, use, and sequestration; (iii) industrial symbiosis and circularity; (iv) urban energy systems’ integration; (v) waste management; (vi) power-to-x-to-power; (vii) seasonal energy storage, and (viii) integration to gas and electricity services.

Evaluating the performance of energy conversion processes requires defining sustainable metrics that ensure objective and reproducible assessments, thus enabling a rational basis for assessing innovative integrated concepts on carbon mitigation, electrification, storage, and renewable energy use. In this way, thermodynamic integration and optimization approaches must ensure that any improvement on an industrial system does not trigger a socio-economic or environmental issue elsewhere (burden-shifting). Thus, although biomass energy utilization for fuels’ and chemicals’ production can be generally seen as a renewable option [2], its use must be carefully examined to certify that the energy technologies and systems involved are compatible with the sustainable energy transition scenarios. For this reason, process integration and optimization have become more and more relevant to assess the performance of biomass energy-based sectors, such as biorefineries. The implementation of carbon capture and storage has gained recent attention as it could lead to net-negative CO_2_ emissions. It explains why innovative designs are being explored to make the best use of biomass resources, while observing their sustainability targets.

Meanwhile, systems like biomass-integrated gasification combined cycles (BIGCCs), featuring organic Rankine cycles and absorption refrigeration cycles, aim to demonstrate the potential of the multi-process and multi-product approaches in which waste heat becomes an asset rather than an industrial burden [3]. In the same way, the advanced exergy-based optimization of polygeneration systems using non-conventional working fluid aims to offset the concerns of conventional water-based Rankine cycles, especially in terms of footprint and water scarcity impacts. A high degree of self-power generation of industrial and chemical processes presents new opportunities for electrification efforts towards an industrial heating decarbonization [4]. Novel high-temperature heat pump technologies are being developed to upgrade the low-temperature waste heat available in industrial sites by consuming only a fraction of the high-grade energy input that would be needed if fossil fuels were used instead [5].

Some emerging technologies, such as machine learning for predicting waste heat availability or the application of artificial neural networks for optimizing equipment performance, demonstrate that the novel paradigms of data-driven thermodynamic process optimization have become prevalent [6]. The massification of these powerful computational tools is expected to unveil breakthroughs in energy systems, like unconventional solar power systems and plasma applications for industrial heating electrification [7]. In brief, by combining technical, economic, and sustainability perspectives, this Special Issue aims to bring together different research applications to highlight the role of recent advancements in shaping future energy systems (Figure 1).

This Special Issue, entitled “Thermodynamic Optimization of Industrial Energy Systems”, has featured eleven (11) articles on the topics of thermodynamic process design, integration and optimization, leveraging different theoretical and computational methods and tools. Their main contributions are highlighted below:

1. “Advanced Exergy-Based Optimization of a Polygeneration System with CO_2_ as Working Fluid” by Jing Luo, Qianxin Zhu and Tatiana Morosuk [9] focuses on optimizing a polygeneration system using CO_2_ as the working fluid to produce electricity, refrigeration and heating. Advanced exergy-based methods are implemented, splitting exergy destruction into avoidable and unavoidable parts to identify improvement priorities. Exergoeconomic graphical optimization is conducted at the component level, enhancing system performance. The findings highlight the system potential for energy efficiency and cost-effectiveness, with a 15.4% increase in exergetic efficiency and a 7.1% cost reduction.
2. “Optimization and Tradeoff Analysis for Multiple Configurations of Bio-Energy with Carbon Capture and Storage Systems in Brazilian Sugarcane Ethanol Sector” by Bruno Bunya, César A. R. Sotomonte, Alisson Aparecido Vitoriano Julio, João Luiz Junho Pereira, Túlio Augusto Zucareli de Souza, Matheus Brendon Francisco and Christian J. R. Coronado [10] analyzes bio-energy systems with carbon capture and storage (BECCS) in sugarcane ethanol plants. Some cogeneration setups with chemical absorption were evaluated. A single regenerator system reportedly captures the most CO_2_ (51.9%), but reduces plant efficiency by 14.9%. Achieving higher CO_2_ capture rates can lead to higher specific emissions (gCO_2_/kWh) compared to a base plant. Systems with lower CO_2_ capture rates (<51%) ensure overall emission reductions.
3. “Exergoeconomic Analysis and Optimization of a Biomass Integrated Gasification Combined Cycle Based on Externally Fired Gas Turbine, Steam Rankine Cycle, Organic Rankine Cycle, and Absorption Refrigeration Cycle” by Jie Ren, Chen Xu, Zuoqin Qian, Weilong Huang and Baolin Wang [11] explores a novel biomass-based combined cooling and power system. The system integrates an externally fired gas turbine, steam and organic Rankine cycles, and absorption refrigeration cycles. The analysis reveals a thermal efficiency of 70.67%, an exergy efficiency of 39.13%, and a levelized cost of exergy of 11.67 USD/GJ. Adjustments in system parameters can achieve a 5.7% LCOE reduction with minor efficiency trade-offs.
4. “Simultaneous Optimization and Integration of Multiple Process Heat Cascade and Site Utility Selection for the Design of a New Generation of Sugarcane Biorefinery” by Victor Fernandes Garcia and Adriano Viana Ensinas [12] addresses the economic and environmental challenges of sugarcane biorefineries through a novel superstructure model. It integrates heat recovery, utility selection and optimal sizing to design efficient biorefinery configurations. Results show an increase in energy efficiency (from 50.25% to 74.5%) by integrating methanol production to the sugarcane biorefinery via bagasse gasification. Similar results were found for DME production, although the higher power consumption from CO_2_ hydrogenation impacts the energy efficiency.
5. “Combining Exergy and Pinch Analysis for the Operating Mode Optimization of a Steam Turbine Cogeneration Plant in Wonji-Shoa, Ethiopia” by Shumet Sendek Sharew, Alessandro Di Pretoro, Abubeker Yimam, Stéphane Negny and Ludovic Montastruc [13] studies the impact of the operating conditions of a steam turbine in an existing cogeneration plant. By combining pinch and exergy analysis, the research highlights the trade-off between heat integration design and exergy losses. The analysis indicates opportunities for heat pump technology integration and energy savings up to 83.44 MW by reducing exergy losses in the cogeneration plant.
6. “Techno–Economic Analysis of the Optimum Configuration for Supercritical Carbon Dioxide Cycles in Concentrating Solar Power Systems” by Rosa P. Merchán, Luis F. González-Portillo and Javier Muñoz-Antón [14] evaluates the techno-economic performance of supercritical carbon dioxide (sCO_2_) cycles in concentrating solar power (CSP) systems, focusing on the trade-offs between cost and efficiency. Key factors such as the turbine inlet temperature, ambient conditions, pressure drops, and turbomachinery efficiency are analyzed, alongside uncertainties in the component and heating costs. The CSP system with partial cooling offers the lowest costs, though under certain conditions, simple cycles or recompression cycles may be more economical.
7. “Exergoeconomic Analysis of a Mechanical Compression Refrigeration Unit Run by an ORC” by Daniel Taban, Valentin Apostol, Lavinia Grosu, Mugur C. Balan, Horatiu Pop, Catalina Dobre and Alexandru Dobrovicescu [15] showcases the exergoeconomic optimization of a vapor compression refrigeration cycle powered by an organic Rankine cycle recovering waste heat from a diesel engine. It focuses on reducing exergy destruction through structural changes, such as preheating the ORC fluid with an internal heat exchanger, improving global exergetic efficiency by 2.03%. Design improvements such as lowering temperature differences in heat exchangers and increasing the compression efficiency can reduce refrigeration unit costs by 59%.
8. “Improved Waste Heat Management and Energy Integration in an Aluminum Annealing Continuous Furnace Using a Machine Learning Approach” by Mohammad Andayesh, Daniel Alexander Flórez-Orrego, Reginald Germanier, Manuele Gatti and François Maréchal [16] focuses on improving energy efficiency in aluminum annealing continuous furnaces to tackle fossil emissions and fuel costs. A heat transfer model based on the machine learning regression of fluid dynamic simulation results predicts the aluminum temperature and heating rates. Two strategies, namely, optimizing furnace temperature profiles and recycling exhaust flue gasses for energy integration, are explored. A maximum reduction in fuel consumption of 20.7% is attained, optimizing energy integration.
9. “Comparative Exergy and Environmental Assessment of the Residual Biomass Gasification Routes for Hydrogen and Ammonia Production” by Gabriel Gomes Vargas, Daniel Alexander Flórez-Orrego and Silvio de Oliveira Junior [17] evaluates the use of biomass for producing hydrogen and ammonia via gasification to reduce fossil fuel consumption. It highlights the environmental and economic potential of converting biomass into valuable fuels with negative carbon emissions. The work reveals exergy inefficiencies from gasification, but also highlights the potential negative emissions for hydrogen (−5.95 kg_CO2_ per kg_H2_) and ammonia (−1.615 kg_CO2_ per kg_NH3_) production.
10. “Modeling and Optimization of Hydraulic and Thermal Performance of a Tesla Valve Using a Numerical Method and Artificial Neural Network” by Kourosh Vaferi, Mohammad Vajdi, Amir Shadian, Hamed Ahadnejad, Farhad Sadegh Moghanlou, Hossein Nami and Haleh Jafarzadeh [18] examines the optimization of a Tesla valve using artificial neural networks. Key geometrical parameters and inlet velocity are used as inputs, whereas the pressure drop ratio and the temperature difference ratio are the outputs. ANN models trained on numerical data achieved high accuracy in predicting responses. The results highlight the potential of the Tesla valve for advanced applications in heat sinks and exchangers.
11. “Design and Performance Evaluation of Integrating the Waste Heat Recovery System (WHRS) for a Silicon Arc Furnace with Plasma Gasification for Medical Waste” by Yuehong Dong, Lai Wei, Sheng Wang, Peiyuan Pan and Heng Chen [19] proposes a waste heat recovery system for a silicon arc furnace with plasma gasification for medical waste treatment (23,040 t/y). The plasma gasifier also disposes of harmful silica particles from polysilicon production. The syngas produced is used to generate power (4.17 MW of power with 33.99% efficiency) and auxiliary heating. An investment of $18.84 million entails a payback period of 3.94 years.

## Figures and Tables

**Figure 1:**
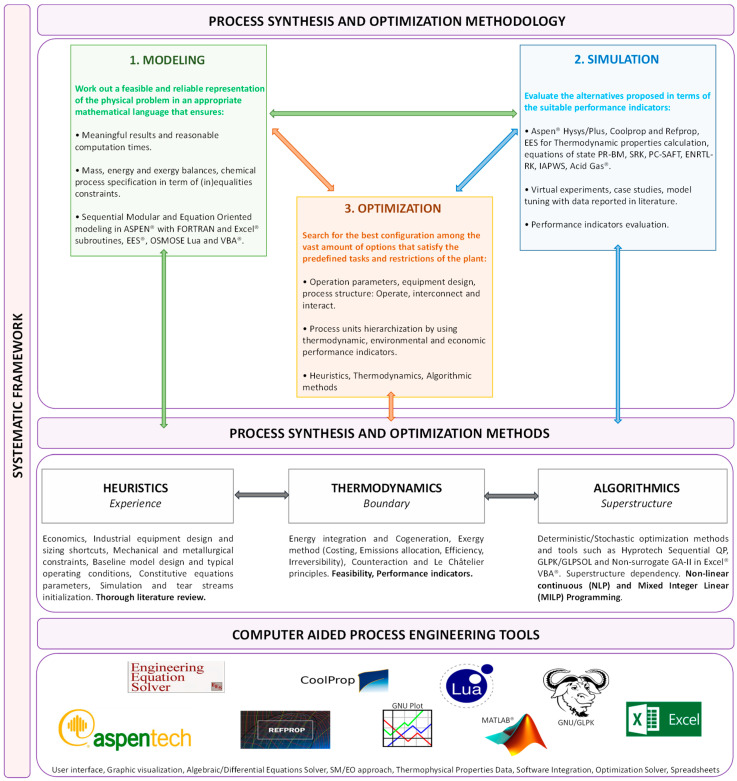
Schematic of thermodynamic optimization and industrial energy systems [8].
